# Magnetostratigraphic dating of the hominin occupation of Bailong Cave, central China

**DOI:** 10.1038/s41598-018-28065-x

**Published:** 2018-06-26

**Authors:** Yanfen Kong, Chenglong Deng, Wu Liu, Xiujie Wu, Shuwen Pei, Lu Sun, Junyi Ge, Liang Yi, Rixiang Zhu

**Affiliations:** 1 State Key Laboratory of Lithospheric Evolution, Institute of Geology and Geophysics, Chinese Academy of Sciences, Beijing, 100029 China; 2 Institutions of Earth Science, Chinese Academy of Sciences, Beijing, 100029 China; 3 College of Earth and Planetary Sciences, University of Chinese Academy of Sciences, Beijing, 100049 China; 4 Institute of Vertebrate Paleontology and Paleoanthropology, Chinese Academy of Sciences, Beijing, 100044 China; 5 State Key Laboratory of Marine Geology, Tongji University, Shanghai, 200092 China

## Abstract

Intermontane basins in the southern piedmont of the Qinling Mountains are important sources of information on hominin occupation and settlement, and provide an excellent opportunity to study early human evolution and behavioral adaptation. Here, we present the results of a detailed magnetostratigraphic investigation of the sedimentary sequence of hominin-bearing Bailong Cave in Yunxi Basin, central China. Correlation to the geomagnetic polarity time scale was achieved using previously published biostratigraphy, ^26^Al/^10^Be burial dating, and coupled electron spin resonance (ESR) and U-series dating. The Bailong Cave hominin-bearing layer is dated to the early Brunhes Chron, close to the Matuyama-Brunhes geomagnetic reversal at 0.78 Ma. Our findings, coupled with other records, indicate the flourishing of early humans in mainland East Asia during the Mid-Pleistocene climate transition (MPT). This suggests that early humans were adapted to diverse and variable environments over a broad latitudinal range during the MPT, from temperate northern China to subtropical southern China.

## Introduction

The chronology of human evolution in different paleoclimatic and paleoenvironmental settings is an intriguing topic in the study of human origins^1–4^. Since the *Homo erectus* remains were excavated at the Zhoukoudian site in Beijing during the 1920s and 1930s^5,6^, numerous hominin/Paleolithic localities were discovered and reported in China, which offer an excellent opportunity to study early human evolution. During the past three decades considerable progress has been made toward dating the stratigraphic record that contains Paleolithic artifacts or hominin fossils in China^1,7–11^, thus significantly contributing to our understanding of early human occupation in mainland East Asia.

**Figure 1:**
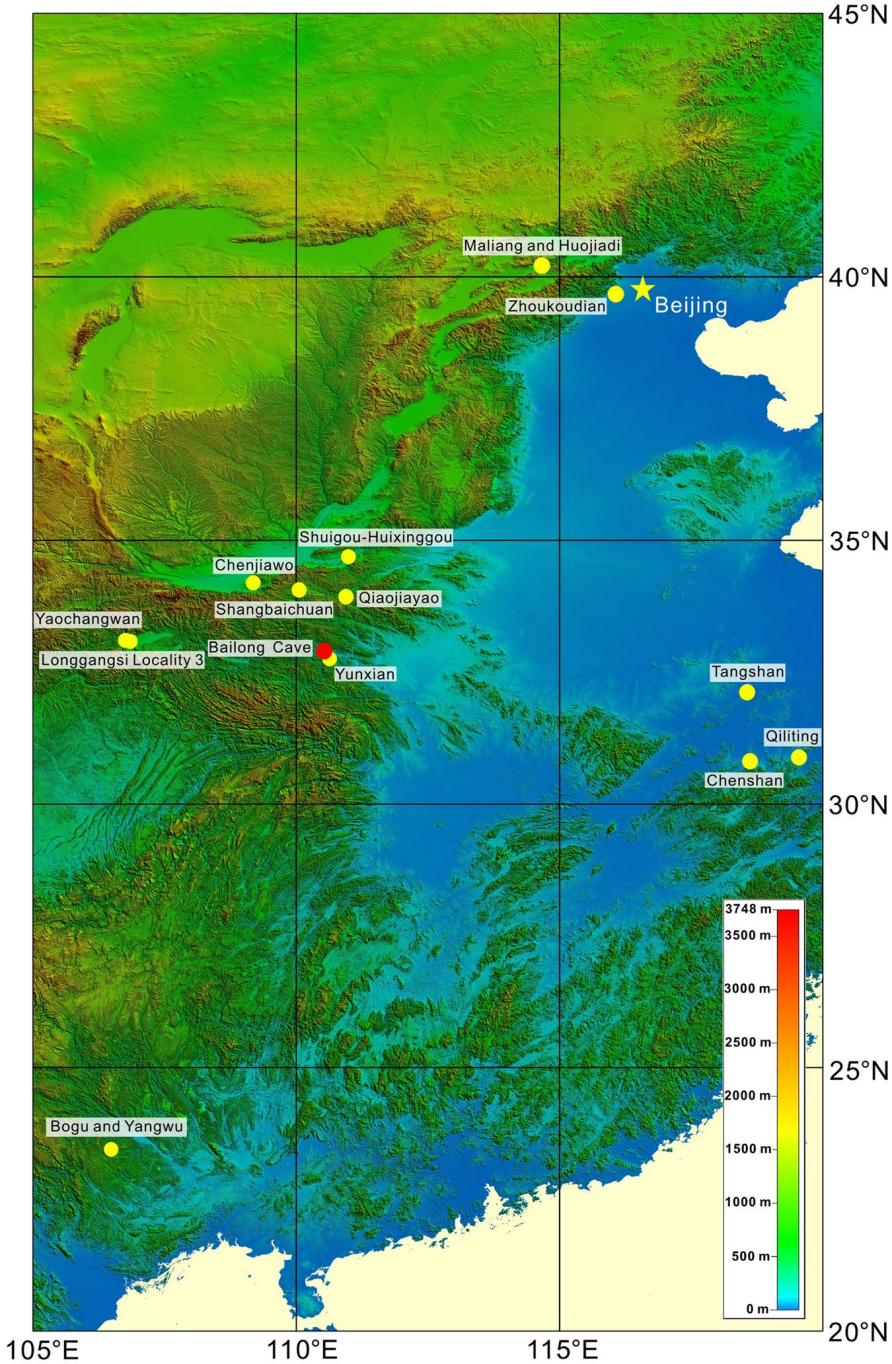
Schematic map of the locations of Bailong Cave (red dot) and other hominin/Paleolithic sites (yellow dots) that date to the Mid-Pleistocene climate transition (1.0–0.6 Ma) in China. The map was generated using DIVA-GIS 7.5 (http://www.diva-gis.org/).

The Qinling Mountains are the traditional dividing line between temperate northern China and subtropical southern China. Significantly, intermontane basins in the southern piedmont of the Qinling Mountains have yielded numerous fossil-containing and archeological localities^12–17^. Thus, the Qinling Mountains and adjacent areas in central China are a key area for studying human occupation in East Asia and for exploring the hominin migration route between southern and northern China^16–20^. Bailong Cave (32°59′40.0′′N, 110°31′33.6′′E, 550 m a.s.l.) is in Shenwuling village, 10 km east of Yunxi County, Hubei Province, central China^21^ (Fig. 1). Here, we present new magnetostratigraphic dating results for the Bailong Cave sedimentary sequence containing hominin teeth^22–27^, which were assigned to *Homo erectus*^25,27^. In addition, combined with a previously published chronology of early humans in mainland East Asia (Table 1), we attempt to provide new insights into early human colonization and adaptability to diverse and variable environments during the Mid-Pleistocene climate transition.

**Table 1:** Hominin/Paleolithic sites during the Mid-Pleistocene climate transition (1.0−0.6 Ma) in China.

Location	Site	Age (Ma)	Dating method	Reference
Nihewan Basin	Maliang	~0.8	Magnetostratigraphy	Wang *et al*.^59^
Nihewan Basin	Huojiadi	~1.0	Magnetostratigraphy	Liu *et al*.^60^
Beijing	Zhoukoudian	0.77 ± 0.08	^26^Al/^10^Be burial	Shen *et al*.^11^
Sanmenxia Basin	Shuigou-Huixinggou	~0.9	Magnetostratigraphy	Li *et al*.^61^
Lantian	Chenjiawo	~0.65	Magnetostratigraphy	An and Ho^18^
Luonan Basin	Shangbaichuan	~0.8	Magnetostratigraphy	Lu *et al*.^13^
Lushi Basin	Qiaojiayao	0.6−0.62	Magnetostratigraphy and OSL	Lu *et al*.^12^
Hanzhong Basin	Yaochangwan	~0.6	Magnetostratigraphy and OSL	Sun *et al*.^14^
Hanzhong Basin	Longgangsi locality 3	~0.9	Magnetostratigraphy	Sun *et al*.^16^
Yunxian	Yunxian Man	~0.9	Magnetostratigraphy	Yan^62^
		~1.1	ESR and ESR/U-series	Bahain *et al*.^63^
Changxing	Qiliting	~1.0	Magnetostratigraphy	Liu *et al*.^64^
				Liu and Deng^65^
Nanjing	Tangshan	0.577 + 0.044/−0.034	TIMS U-series	Zhao *et al*.^66^
Xuancheng	Chenshan	~0.8	Magnetostratigraphy	Liu and Deng^65^
				Liu *et al*.^67^
Bose Basin	Bogu and Yangwu	0.803 ± 0.003	^40^Ar/^39^Ar	Hou *et al*.^10^
Yunxi Basin	Bailong Cave	0.509 ± 0.016	ESR/U-series	Han *et al*.^40^
		0.76 ± 0.06	^26^Al/^10^Be burial	Liu *et al*.^39^

## Results

### Mineral magnetism

**Figure 2:**
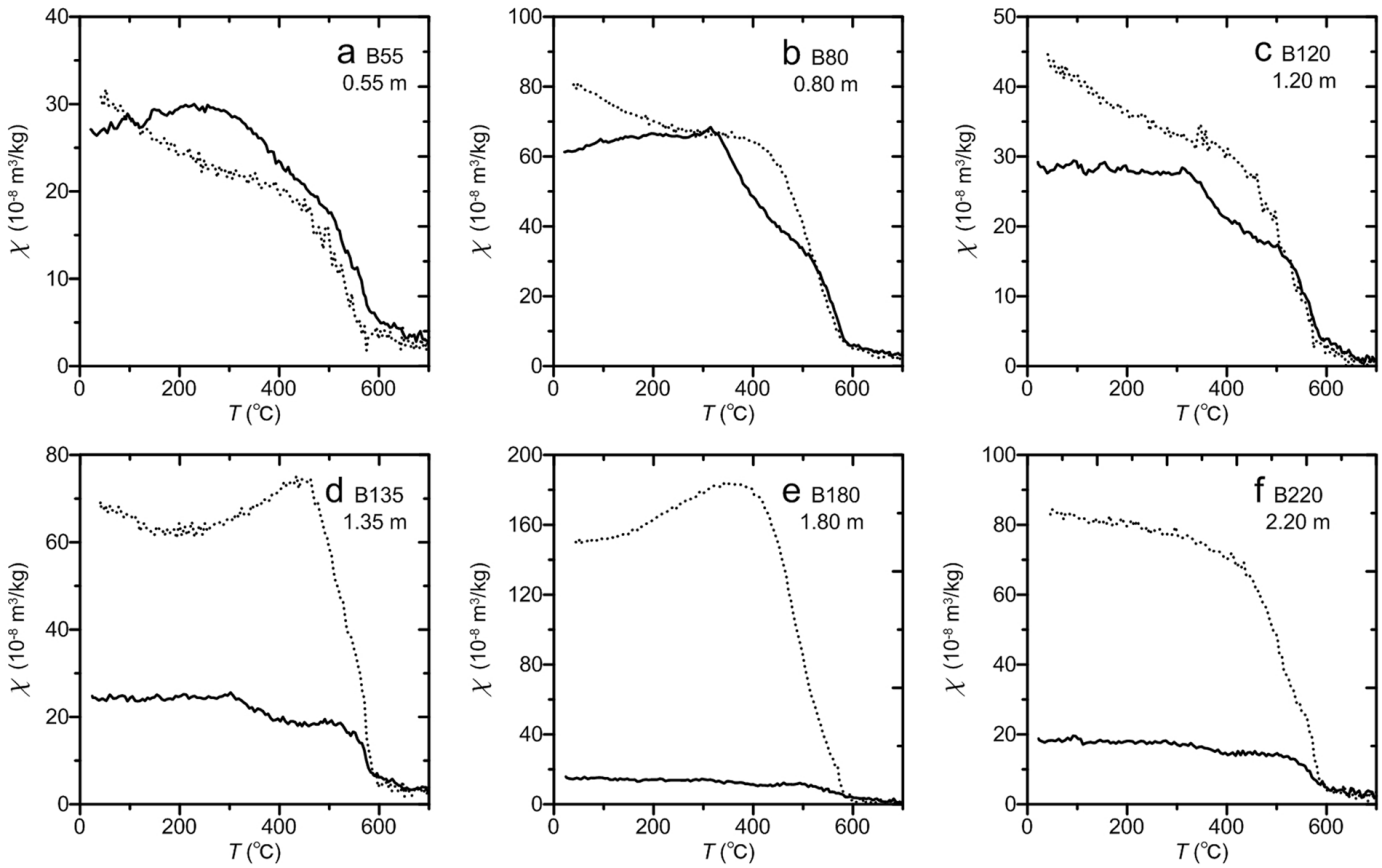
High-temperature magnetic susceptibility (χ-*T* curves) for representative samples. Solid and dotted lines represent heating and cooling curves, respectively.

**Figure 3:**
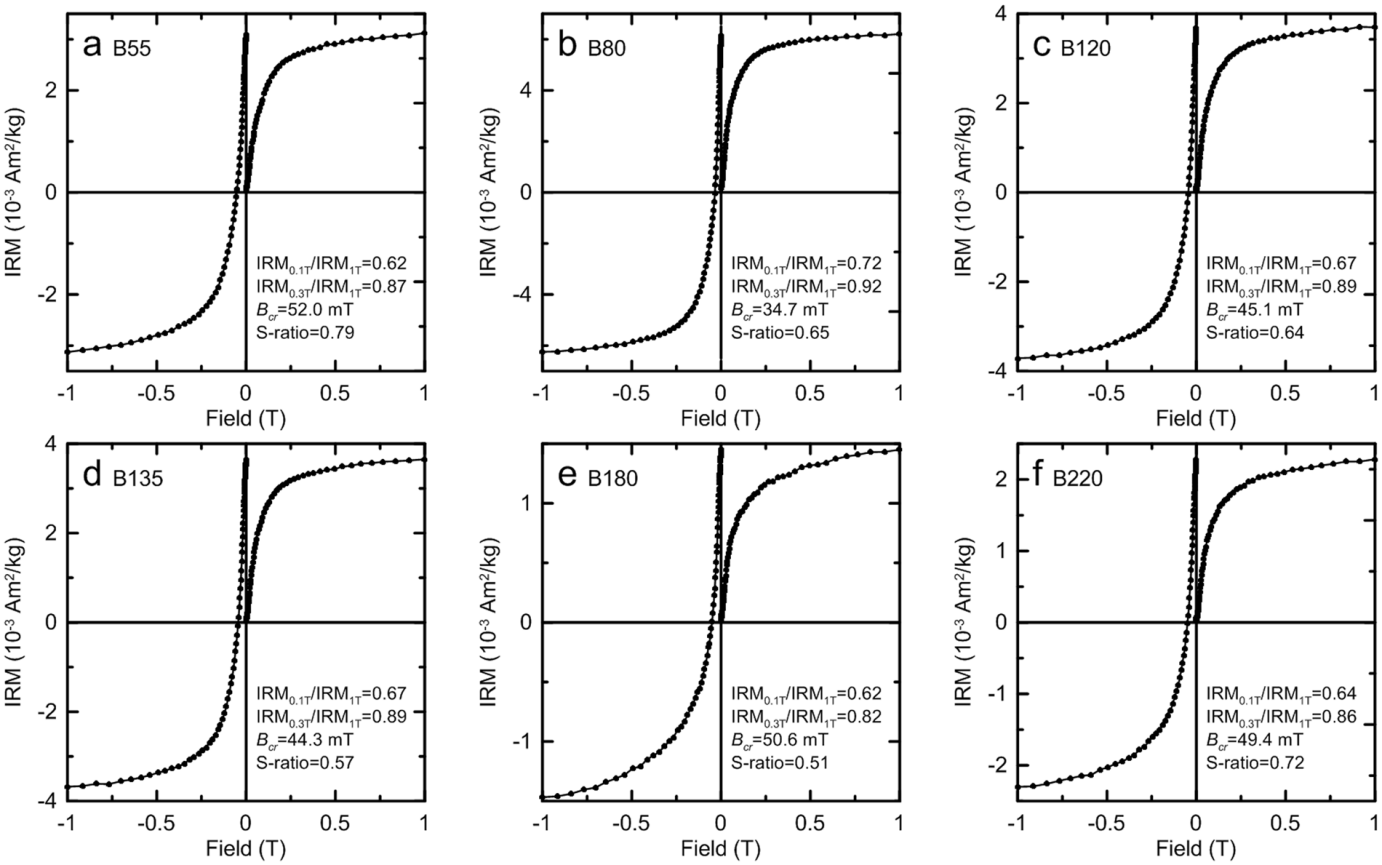
Isothermal remanent magnetization (IRM) acquisition curves and backfield demagnetization curves. Relevant magnetic parameters are indicated.

**Figure 4:**
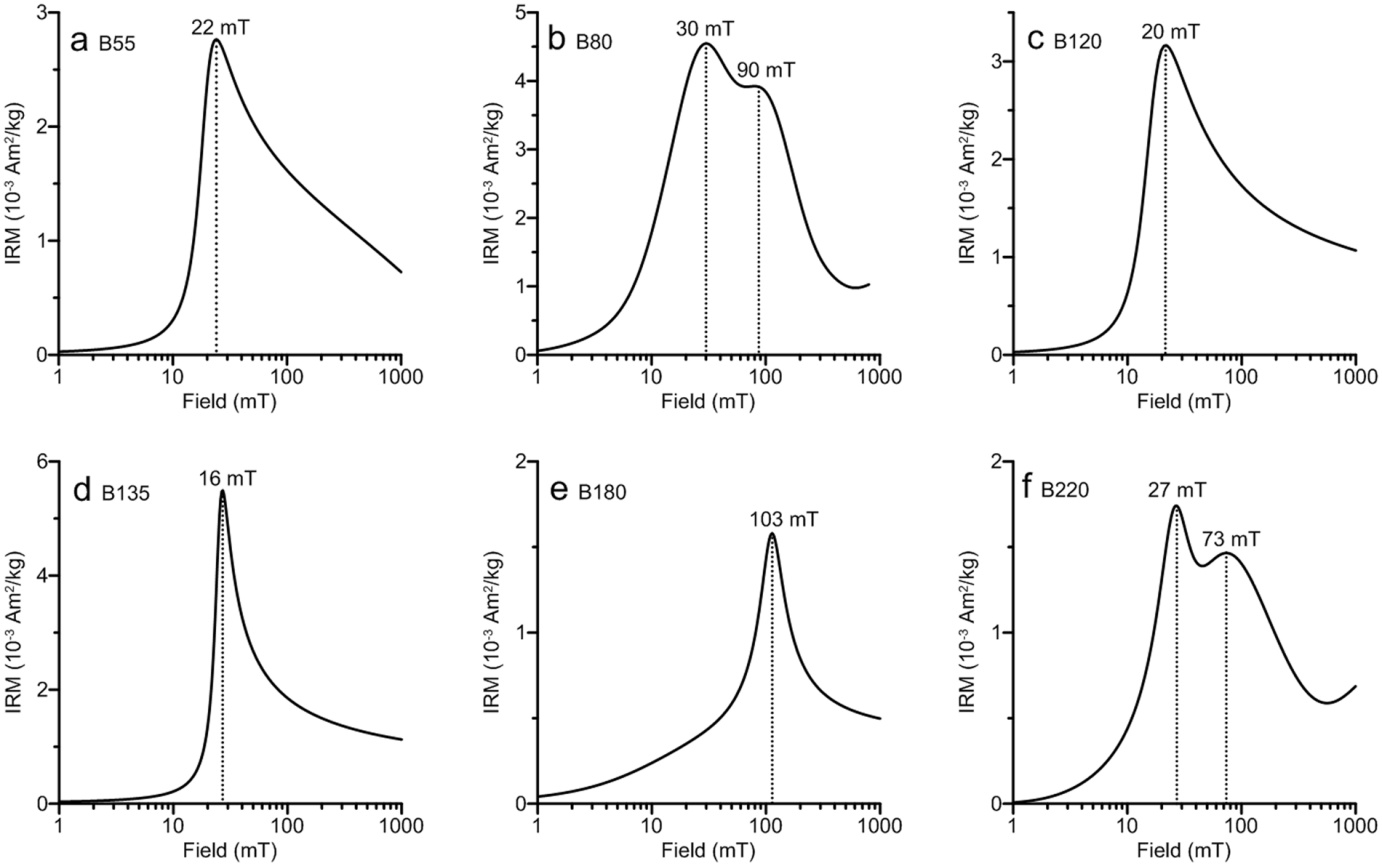
Coercivity distributions for representative samples, calculated with the MAG-MIX package of Egli^32^ (http://dourbes.meteo.be/aarch.net/onlytxt/magmix.otxt_en.html). Coercivity peaks are indicated.

**Figure 5:**
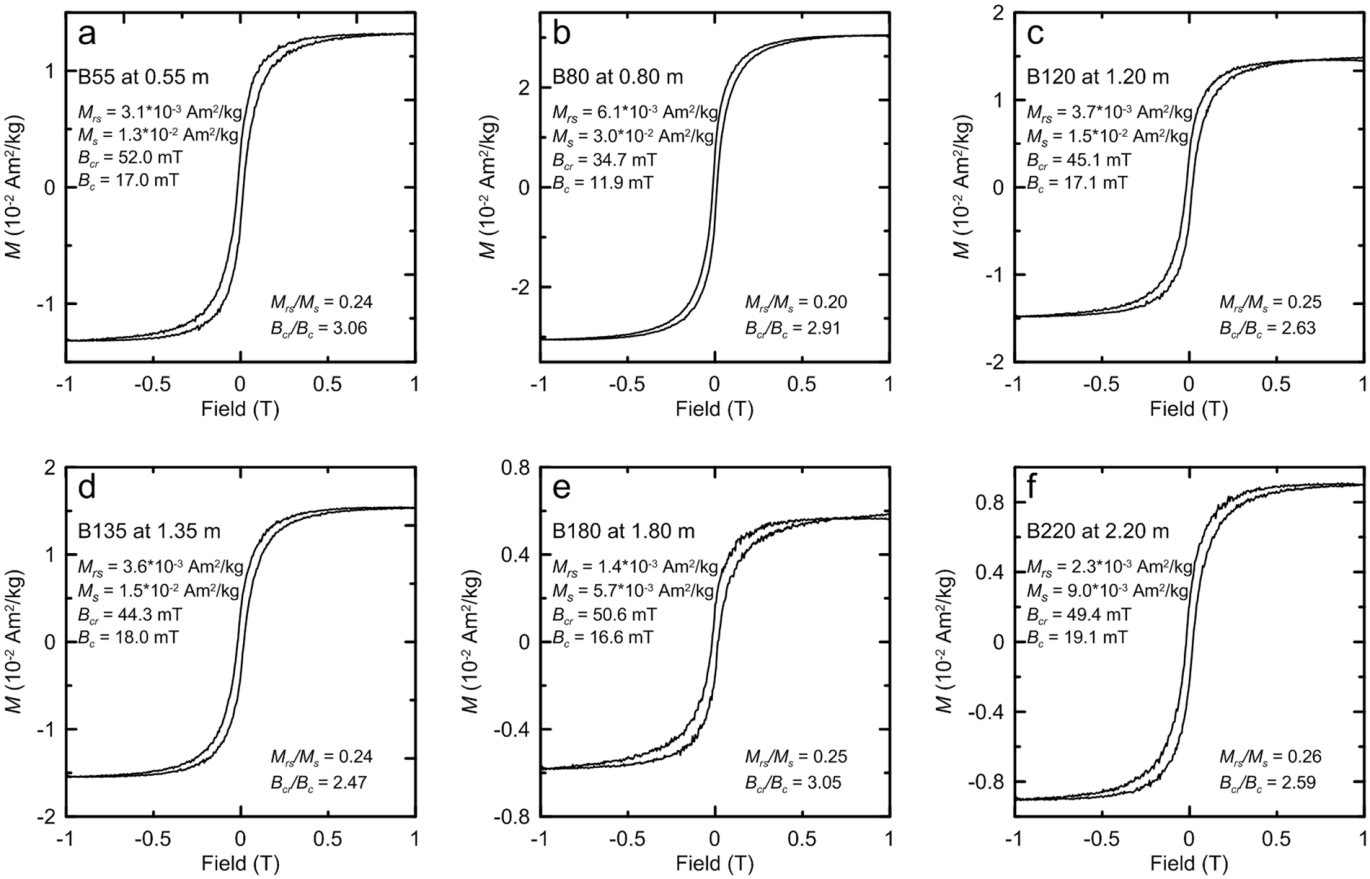
Hysteresis loops after high-field slope correction. Hysteresis parameters are indicated.

**Figure 6:**
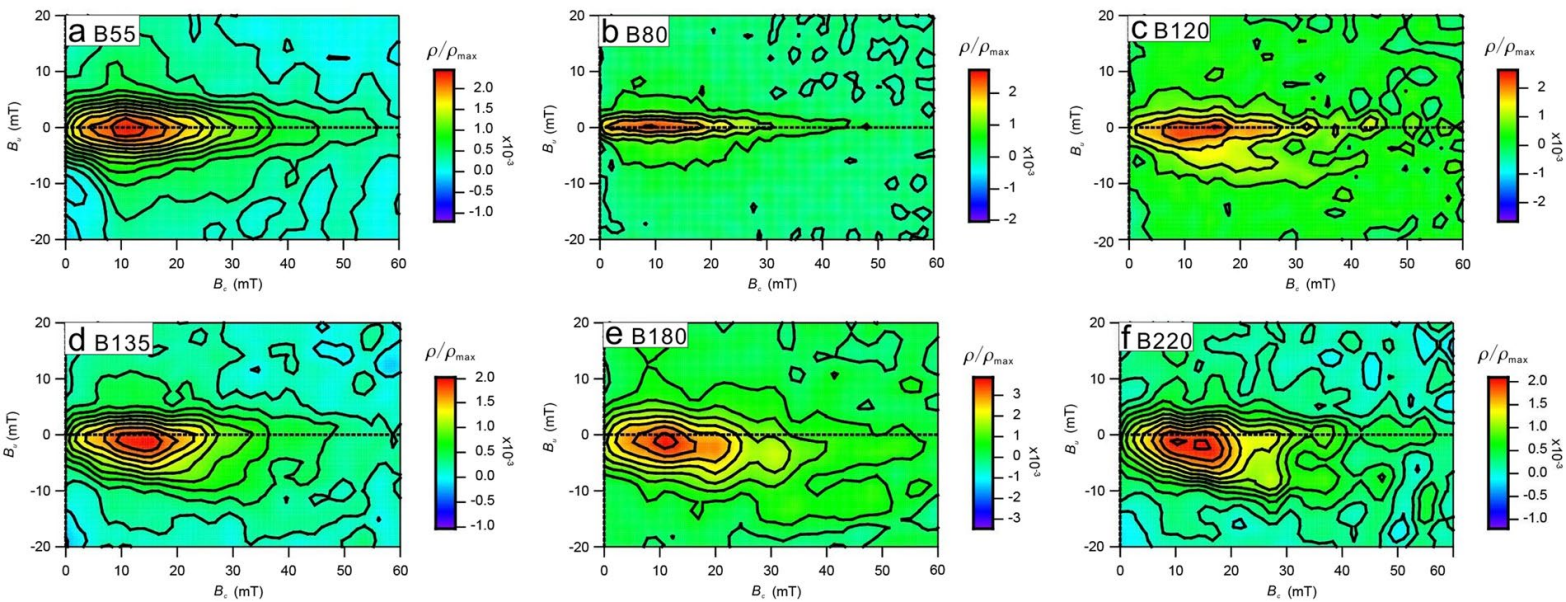
First-order reversal curve (FORC) diagrams for representative samples, which are calculated with a smoothing factor of 5 (**a**,**d**,**e**,**f**) or 3 (**b**,**c**) using the FORCinel software package of Harrison and Feinberg^55^ (https://earthref.org/FORCinel/).

The results of mineral magnetic measurements are illustrated in Figs 2–6. Temperature-dependent magnetic susceptibilities (χ-*T* curves) are sensitive to mineralogical changes during thermal treatment, which can provide information about magnetic mineral composition^28,29^. All of the measured χ-*T* curves (Fig. 2) are characterized by a major susceptibility decrease at about 585 °C, the Curie point of magnetite, which indicates that magnetite is the major contributor to the susceptibility. For some samples, there is a magnetic susceptibility decrease between ~300 °C and ~450 °C in the heating curves (Fig. 2b–d), which is due to the conversion of metastable maghemite to hematite^30^. Two types of χ-*T* curves are evident. One type has cooling curves that are much higher than the heating curves, with susceptibility increasing significantly on cooling below ~585 °C (Fig. 2c–f). The significantly enhanced susceptibility after thermal treatment may arise from the neo-formation of magnetite grains from iron-containing silicates/clays, or from the formation of magnetite by reduction in the presence of combusting organic matter^29,31^. The other type has slightly enhanced susceptibility when cooling to room temperature (Fig. 2a,b).

**Figure 7:**
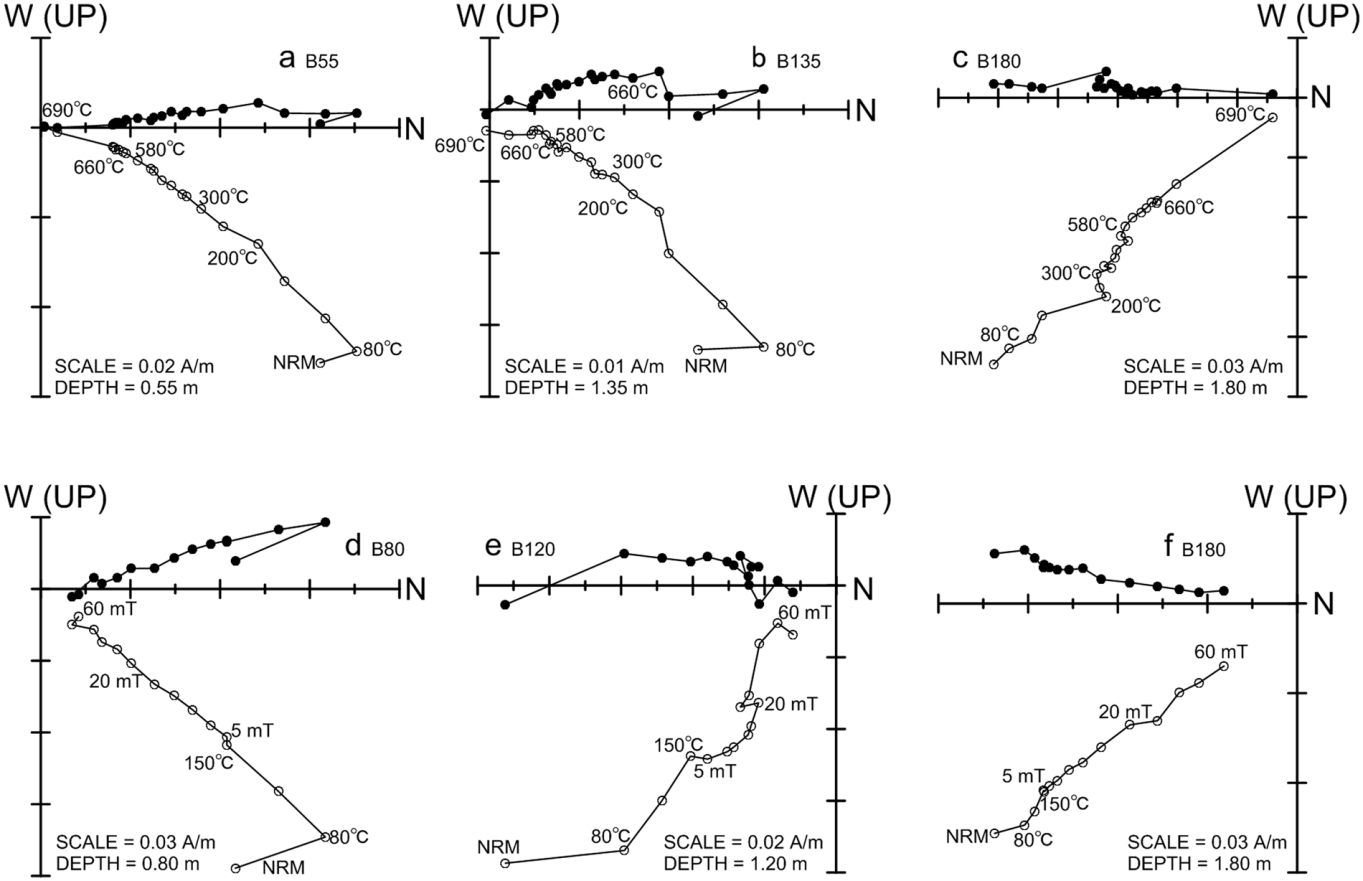
Orthogonal projections of stepwise thermal and alternating field demagnetization data. The solid and open circles represent projections onto the horizontal and vertical plane, respectively. The numbers refer to temperatures in °C or alternating fields in mT. NRM is the natural remanent magnetization.

These χ-*T* curves provide further evidence that magnetite and maghemite are the dominant ferrimagnetic minerals in the Bailong Cave deposits. Hematite, which is another important carrier of the natural remanence suggested by isothermal remanent magnetization (IRM) acquisition (Figs 3 and 4), hysteresis loops (Fig. 5), and progressive thermal demagnetization analyses (Fig. 7), is not well expressed in the χ-*T* curves because its weak susceptibility is masked by the much stronger contributions of magnetite and/or maghemite.

IRM acquisition and backfield demagnetization curves provide information about the coercivity (*B*_c_) distribution and coercivity of remanence (*B*_cr_), which can help to discriminate magnetic phases with different values of *B*_c_ and *B*_cr_^32^. All the selected samples have similar IRM curves (Fig. 3). The rapid increase in the IRM acquisition curves below 100 mT indicates the dominant presence of magnetically soft components, such as magnetite and maghemite. However, the IRM of all the samples continues to increase above 300 mT, and the S-ratio^33^, which is defined as the ratio of IRM acquired at −0.3 T (IRM_−0.3T_) to IRM acquired at 1 T (IRM_1T_), has relatively low values (generally below 0.8) (Fig. 3). This behavior suggests a significant contribution from high-coercivity minerals (hematite) which have a weak magnetization. Unmixing methods^32^ were used to analyze the magnetic mineral composition. Derivatives of the IRM acquisition curves are plotted to illustrate the coercivity distributions (Fig. 4), where one- to two-humped distributions illustrate distinct coercivity distributions with peaks at 20–30 mT and ~100 mT. The lower coercivity component is likely to be magnetite and/or maghemite, and the higher coercivity component represents hematite.

Hysteresis loops^28^ and first-order reversal curve (FORC) diagrams^34,35^ provide information about the coercivity spectrum and domain state of ferrimagnetic materials. All the selected samples have wasp-waisted hysteresis loops (Fig. 5), which are attributed to the coexistence of two magnetic components with strongly contrasting coercivities^28^. The low-coercivity component consists of magnetite and/or maghemite, and the high-coercivity component is mainly due to hematite, as suggested by the χ-*T* curves (Fig. 2) and progressive thermal demagnetization analyses (Fig. 7). FORC diagrams were obtained to provide a more detailed interpretation of the domain state of magnetic mineral assemblages. All samples have FORC distributions that are indicative of stable single-domain (SD) particles (Fig. 6). The vertical spread along the *B*_c_ axis is mostly ~20 mT. The FORC diagram for sample B80 (Fig. 6b) suggests a low degree of magnetostatic interactions, as indicated by a ridge-like distribution along the *B*_c_ axis, which suggests the dominance of non-interacting SD particles. All FORC diagrams are centered on the *B*_c_ axis at 10–20 mT, which is consistent with the dominance of magnetite.

### Paleomagnetic measurements

**Figure 8:**
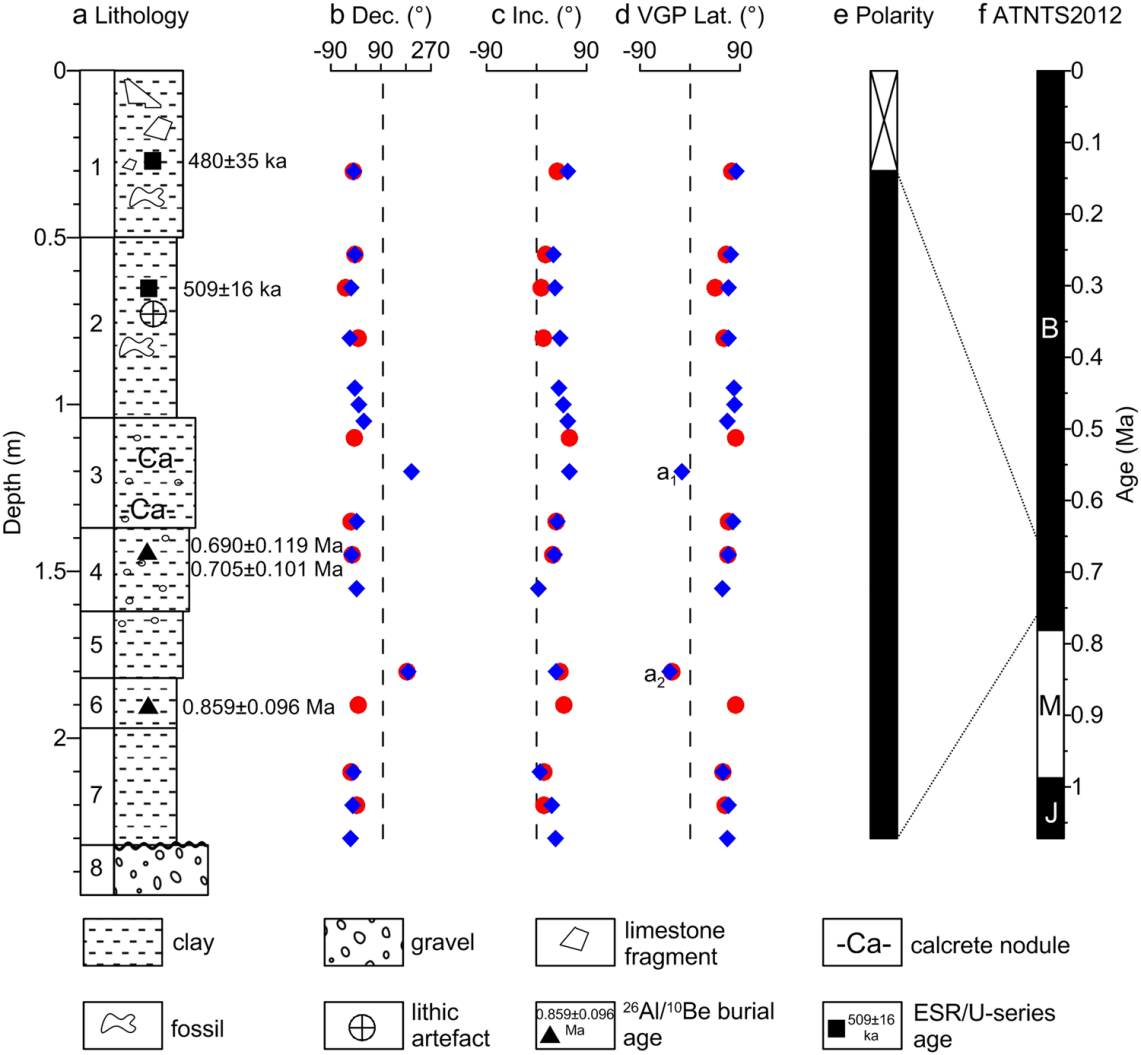
Lithostratigraphy (**a**) and magnetic polarity stratigraphy (**b–e**) of the sedimentary sequence at Bailong Cave, and correlation with the astronomically tuned Neogene timescale of Hilgen *et al*.^56^ (ATNTS2012) (**f**). ^26^Al/^10^Be burial and ESR/U-series ages respectively reported by Liu *et al*.^39^ and Han *et al*.^40^ are shown in (**a**). The cross on the top of the polarity column (**e**) indicates that no samples were taken from this interval. Red circles and blue diamonds in (**b–d**) represent thermally and AF demagnetized specimens, respectively. a1 and a2 in (**d**) represent anomalous paleomagnetic directions. Dec., declination; Inc., inclination; MAD, maximum angular deviation; VGP Lat., the virtual geomagnetic pole latitude; B, Brunhes; M, Matuyama; J, Jaramillo.

The characteristic remanent magnetization (ChRM) was isolated after removal of one or two soft secondary magnetization components (Fig. 7). Principal component analysis (PCA) was performed on stepwise demagnetization data using the PaleoMag software^36^. The principal component direction was computed using a least-squares fitting technique^37^. Demagnetization results for representative specimens, as shown in orthogonal diagrams^38^, indicate that both magnetite and hematite dominate the remanence, because a high-stability ChRM component persists up to 690 °C (Fig. 7a–c) or up to 60 mT (Fig. 7d–f). After the combined thermal and alternating field (AF) demagnetization, or thermal demagnetization only, 15 out of 18 and 11 out of 12 specimens with maximum angular deviation (MAD) values <15° yielded reliable ChRM directions, respectively. The ChRM vector directions yielded virtual geomagnetic pole (VGP) latitudes that were used to define the magnetostratigraphic polarity succession for the Bailong Cave section. A single, normal polarity zone is recognized (Fig. 8). In addition, two specimens recorded negative VGP latitudes, labeled a1 and a2 in Fig. 8d. These two anomalous paleomagnetic directions could represent short-period geomagnetic variations; however, we exclude them as possible geomagnetic excursions because they are based on a single specimen only.

## Discussion

### Chronology of the Bailong Cave sedimentary sequence and age estimation of hominin occupation

We established the chronology of the Bailong Cave hominin-bearing sequence by combining the previously published biochronology^26^, ^26^Al/^10^Be burial dating^39^ and coupled ESR/U-series dating results^40^ with our new magnetochronology.

Three excavations at Bailong Cave, in 1977, 1982, and 2007–2009, yielded abundant mammalian fossils. Five orders of mammals were identified by Wu *et al*.^26^, including rodentia, carnivora, proboscidea, perissodactyla, and artiodactyla, comprising 29 taxa, as listed in Table 2. The mammalian fossils include typical species of the *Stegodon-Ailuropoda* fauna *sensu lato*^41,42^, such as *Rhizomys* sp., *Hystrix* sp., *Stegodon* sp., *Aliuropoda wulingshanensis*, *Rhinoceros sinensis*, *Tapirus sinensis*, *Cervus yunnanensis*, *Capricornis sumatraensis*, *Nemorhaedus* sp., and *Bubalus* sp. The *Stegodon-Ailuropoda* fauna are dated from the late Early to Late Pleistocene^41^. However, archaic taxa such as *Cuon javanicus*, *Pachycrocuta licenti*, *Aliuropoda wulingshanensis*, *Sivapanthera pleistocaenicus*, *Megantereon* sp., *Sus peii*, *Cervavitus fenqii*, *Cervus elegans*, *Cervus yunnanensi*s, and *Leptobos brevicornis*, led Wu *et al*.^26^ to conclude that the Bailong Cave fauna may be no younger than the early Middle Pleistocene. Recently, Dong^43^ assigned the Bailong Cave fauna to the 500–850 ka age range according to fauna antiquity coefficients, faunal binary similarity coefficients, faunal extinction rates, and ecological composition similarities of 15 hominin-bearing faunal sites in China.

**Table 2:** List of mammalian fauna in Bailong Cave (after Wu *et al*.^26^).

Taxon
*Homo erectus*
*Rhizomys* sp.
*Hystrix* sp.
*Stegodon* sp.
*Cuon javanicus*
*Pachycrocuta licenti*
*Aliuropoda wulingshanensis*
*Arctonyx collaris*
*Paguma larvata*
*Viverra* sp.
*Ursus* sp.
*Panther pardus*
*Sivapanthera pleistocaenicus*
*Megantereon* sp.
*Felis* sp.
*Panthera tigris*
*Rhinoceros sinensis*
*Tapirus sinensis*
*Sus peii*
*Muntiacus* sp.
*Moschus* sp.
*Cervavitus fenqii*
*Cervus elegans*
*Cervus yunnanensis*
*Capricornis sumatraensis*
*Nemorhaedus* sp.
*Megalovis guangxiensis*
*Budorcas* sp.
*Leptobos brevicornis*
*Bubalus* sp.

Importantly, ^26^Al/^10^Be burial dating^39^ of quartz samples from layers 4 and 6 in the lower part of the Bailong Cave sequence (Fig. 8) give a weighted mean burial age of 0.76 ± 0.06 Ma. Liu *et al*.^39^ further concluded that cultural deposits at Bailong Cave site should be somewhat younger than the above date by considering possible biases introduced by the dating method, stratigraphic order, and the documented rapid sedimentation. Most recently, coupled ESR/U-series dating^40^ was conducted on the fossil teeth of herbivores (Cervidae and Bovidae) from layers 1 and 2 in the upper part of the sequence, yielding a weighted mean age of 509 ± 16 ka for five fossil teeth from layer 2 (Fig. 8a). The ^26^Al/^10^Be burial and ESR/U-series ages, respectively obtained by Liu *et al*.^39^ and Han *et al*.^40^, are within the Brunhes Chron, which provides stringent age control for the Bailong Cave sedimentary sequence. Given the robust chronological constraints from mammalian biochronology^26,43^, ^26^Al/^10^Be burial dating^39^ and ESR/U-series dating^40^, the normal polarity magnetozone identified here in the Bailong Cave sequence must correlate with the early Brunhes Chron, which is close to the Early/Middle Pleistocene transition.

### Geochronological implications

Bailong Cave is a Paleolithic hominin site in an intermontane basin along the Hanjiang River in the southern piedmont of the Qinling Mountains. Available chronological data from a combination of detailed magnetostratigraphic analysis, optically stimulated luminescence dating, and pedostratigraphic correlation with well-dated loess-paleosol sequences of the central Chinese Loess Plateau indicate that hominins occupied the Hanjiang valley several times during the interval from 1.2–0.1 Ma^12–17^. Given the recognition of numerous Paleolithic sites on both the northern and southern sides of the Qinling Mountains, Sun *et al*.^16^ proposed that the Hanjiang River valley was a probable hominin migration route through the Qinling Mountains between subtropical southern China and temperate northern China.

**Figure 9:**
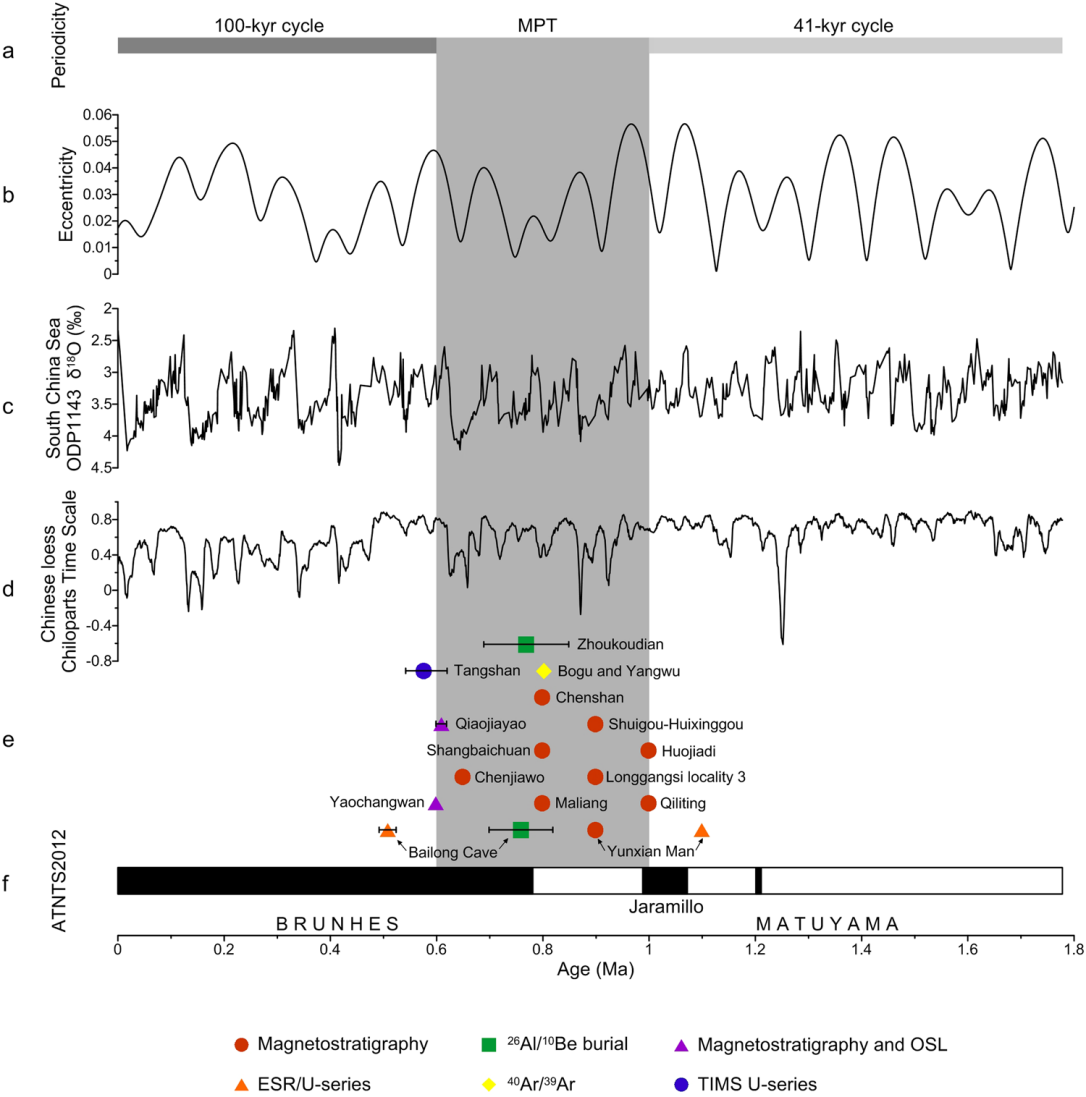
Synthesis of well-dated hominin or Paleolithic sites in China during the Mid-Pleistocene climate transition (MPT) (1.0–0.6 Ma)^46,47^ with respect to ATNTS2012^56^, and temporal variations of both marine and terrestrial paleoclimatic proxies in East Asia. (**a**) Paleoclimatic periodicities. (**b**) Long-term variations of eccentricity^57^. (**c**) δ^18^O record from ODP Site 1143, South China Sea^58^. (**d**) Chinese loess Chiloparts time scale, which is the stacked grain-size age model for Chinese loess/paleosol sequences^48^. (**e**) Paleolithic or hominin sites (see Table 2). (**f**) ATNTS2012^56^. The shaded area represents the MPT, which began at about 1–0.8 Ma and terminated at about 0.7–0.6 Ma^46,47^.

Moreover, by ~1 Ma hominins (mostly *Homo erectus*) occupied a broad latitudinal range in North Africa, Europe, western Asia, and eastern Asia^3,44,45^, which indicates that early human populations had adapted to diverse climatic settings. We note especially that during the Mid-Pleistocene climate transition, which began at about 1.0–0.8 Ma and terminated at about 0.7–0.6 Ma^46,47^, early human populations had flourished and expanded in mainland East Asia, from the low latitudes of the Tropic of Cancer (e.g., the Bose Basin) to high northern latitudes (e.g., the Nihewan Basin) (Figs 1 and 9, Table 1).

From pre- to post-MPT, the dominant periodicity of high-latitude climate oscillations changed from 41 kyr to 100 kyr, leading to profound changes in the length and intensity of glacial-interglacial cycles^46–49^. The MPT was characterized by variable environments^50^, during which the increasing climate contrast between glacial and interglacial periods may have forced early humans to become increasingly resilient to glacial-interglacial cycling^51^.

## Methods

### Geological setting

Bailong Cave (32°59′40.0′′N, 110°31′33.6′′E, elevation 550 m) (Fig. 1) is situated on the northwestern margin of the Wudang Uplift in the Qinling Orogenic Belt. Mesoproterozoic metamorphic volcanic and sedimentary rocks, comprising the Wudangshan Group and Neoproterozoic carbonate and sedimentary rocks comprising the Yaolinghe, Doushantuo, and Dengying Formations, occupy a large part of the area. The Neoproterozoic carbonate rocks form a karst topography controlled by the regional hydrologic system. The Neogene Shaping Formation consists of conglomerate and conglomeratic mudstones, and Quaternary sedimentary deposits unconformably overlie Mesoproterozoic and Neoproterozoic strata^52^.

Bailong Cave developed in the Neoproterozoic carbonate and Neogene sedimentary rocks. The cave deposits are divided lithostratigraphically into 8 sedimentary layers (Fig. 8), which were described in detail by Wu *et al*.^26^ and Dong^43^. Layer 2 is fossiliferous and mainly composed of brownish-red clay with occasional calcareous concretions and gravels (Fig. 8). Eight hominin teeth and associated mammalian fossils and stone artifacts were unearthed from this layer^26,27,53,54^.

### Archeological setting

Bailong Cave archeological site was discovered in 1976 and three systematic excavations were conducted subsequently in 1977, 1982, and 2007–2009. So far, 29 taxa of vertebrate mammals (Table 1) and 38 stone artifacts were unearthed, which were reported in detail by Wu *et al*.^26^ Importantly, 8 hominin teeth, which were assigned to *Homo erectus*^25,27^, were recovered from the Bailong Cave, including two found by farmers in 1976, four by excavation in 1977, one by excavation in 1982, and one by excavation in 2008^27^. The Bailong Cave lithic assemblage is essentially an Oldowan-like industry (i.e., Mode 1 core and flake technologies). Like other Oldowan-like industries in China, the Bailong Cave stone assemblage is characterized by a simple technological design, a low degree of standardization, and casually retouched flakes. Technologically, the Bailong Cave lithic assemblage includes 4 cores, 4 flakes, 10 retouched tools, and 20 chunks and debris fragments. The utilized stone raw material is primarily vein quartz, which can be obtained from local Precambrian outcrops. The principal flaking technique was simple direct hard hammer percussion, followed by bipolar percussion. The cores were moderately exploited, probably due either to the difficulties of flaking low-quality vein quartz, or to the short distance of these rocks to the hominin site^26^.

### Sampling

Due to possible disturbance, the uppermost 0.3 m of the cave sedimentary sequence was removed before sampling. A total of 18 oriented block samples were collected with a magnetic compass at 5–25 cm stratigraphic intervals. Cubic specimens with dimensions of 20 mm × 20 mm × 20 mm were obtained from those block samples in the laboratory.

### Mineral magnetic measurements

Το determine the magnetic mineralogy, a total of 6 representative samples were selected for mineral magnetic measurements, including χ-*T* curves, IRM acquisition curves, backfield IRM demagnetization curves, hysteresis loops, and FORC diagrams.

χ-*T* curves were obtained by continuous exposure of samples through temperature cycles from room temperature to 700 °C and back to room temperature with a ramping rate of 2 °C/min, using an AGICO MFK1-FA equipped with CS-3 temperature control system. To minimize the possibility of oxidation, the samples were heated and cooled in an argon atmosphere. For each sample, we subtracted the contribution of the sample holder and thermocouple to the magnetic susceptibility.

Hysteresis loops, IRM acquisition, back-field demagnetization curves, and FORCs were measured with a Princeton Measurements Corporation MicroMag 3900 vibrating sample magnetometer (VSM) up to a maximum field of 1 T. FORC diagrams were calculated using the FORCinel software package^55^. Magnetic components were analyzed using the unmixing programs written by Egli^32^.

### Paleomagnetic measurements

To establish the magnetic polarity stratigraphy, all specimens were subjected to stepwise demagnetization. To confirm the paleomagnetic results, two sets of parallel specimens were measured on the Bailong Cave samples. First, all 18 specimens were subjected to combined thermal and AF demagnetization at a peak field up to 60 mT at 5–10 mT intervals after stepwise thermal demagnetization at 80 °C, 120 °C, and 150 °C, with a Magnetic Measurements thermal demagnetizer with a residual magnetic field less than 10 nT. Then, the second set of 12 parallel specimens was subjected to stepwise thermal demagnetization up to 690 °C (21 steps with 10–50 °C temperature increments). Both methods are capable of isolating the ChRM after removal of a soft secondary component of magnetization. The remanence measurements were made using a 2-G Enterprises Model 760-R cryogenic magnetometer installed in a magnetically shielded space with background field of <300 nT.

### Data availability

The datasets generated and/or analyzed during the current study are available from the corresponding author on request or from the Magnetics Information Consortium (MagIC) database (http://earthref.org/MAGIC).
